# Mediating Pain: Navigating Endometriosis on Social Media

**DOI:** 10.3389/fpain.2022.889990

**Published:** 2022-05-30

**Authors:** Eileen Mary Holowka

**Affiliations:** Department of Communication Studies, Concordia University, Montreal, QC, Canada

**Keywords:** endometriosis, social media, qualitative, chronic pain, information-sharing, patient communities, ethnography, meaning-making

## Abstract

With the rise of social media, many people with endometriosis have turned to platforms such as Facebook and Instagram in the face of lacking care. This qualitative study focuses on why and how people with endometriosis use these platforms. Despite the risks of misinformation and conflict on social media, the results of this research show that many people with endometriosis find these spaces beneficial, particularly for information sharing, social support, representation, and advocacy practices around endometriosis. Using data collected from surveys and interviews, this study reveals that people with endometriosis often use social media to understand, experiment with, and navigate their symptoms and that these efforts deserve recognition by endometriosis researchers and practitioners. This article proposes that, in order to improve future patient-practitioner and patient-researcher relationships for endometriosis, we must understand, not dismiss, the social media practices of those with endometriosis. By understanding how and why patients turn to social media, clinicians and researchers can build toward more patient-oriented futures.

## Introduction

Endometriosis is a chronic inflammatory condition that affects approximately 1 in 10 women and undetermined numbers of transgender, genderfluid, and non-binary people (1, 2). Endometriosis is defined by tissue that is similar to, but distinct from, the lining of the uterus growing outside the uterus and throughout the body. Primary symptoms include chronic pain, pain with sexual intercourse, fatigue, and infertility which can result in negative emotional impacts and an overall decreased quality of life (3–6). Although endometriosis has economic and societal costs similar to other chronic conditions, such as Rheumatoid Arthritis, Type 2 Diabetes, and Chron's disease, it receives disproportionately less funding (7, 8) and the worldwide average delay between start of symptoms and diagnosis is 7.5 years (9).

It is common for people living with endometriosis to experience dismissal and mistreatment associated with a long history of medical discrimination and sexism around chronic pain, particularly pain associated with gender and menstruation (10–13). The contemporary conception of endometriosis is, in the words of Redwine, “trapped by errant words of the past,” and based on outdated conceptions of the disease and patient-blaming dating as far back as 400 BCE (11, 13, 14). Myth and misconceptions continue to haunt the representation of endometriosis today, where it is commonly referred to as “enigmatic” and “puzzling” by medical professionals and patients alike. Although communication about endometriosis has improved over the last 20 years, awareness of the disease, even in symptomatic individuals, is still lacking (15).

In the face of mistreatment, dismissal, long delays, isolation, and lacking resources, many people with endometriosis turn to the Internet and social media for answers and support (16, 17). In 2018, “what is endometriosis?” was the third most trending health-related question on Google and in each month of 2017 there were more than 400,000 Google searches of endometriosis in the United States alone (18, 19). Much of the literature on endometriosis and social media thus far has focused on information-seeking behaviors and the risks of misinformation (16, 17). While misinformation and information-seeking are important aspects of endometriosis-related social media use, there is more to the picture than just this. This article considers the variety of ways that a global, but predominantly North American, community of English-speaking people with endometriosis use social media and what these many practices communicate about their symptoms, desires, and healthcare needs. Although social media can have benefits, it is not an ideal place to navigate a chronic illness. Despite these challenges, many people with endometriosis continue to find value online. Rather than dismissing these social media practices, analyzing them in our research can help break the pattern of patient-blaming in endometriosis care and lead to more patient-oriented futures.

## Theoretical Framework

This research is necessarily multi-disciplinary. Just as proper endometriosis treatment relies on a multidisciplinary team that can address all aspects of the body and mind, research into endometriosis and social media requires not only a media studies perspective, but also a framework informed by affect theory and feminist disability studies. Online endometriosis spaces are complex and messy, full of conflicting information, heightened emotions, and variably moderated spaces. As social science scholar Kate Seear writes in her book on the disease, endometriosis is already “a disease exemplified by an unusually high degree of uncertainty, mess, and contestation” (20). Social media only further contributes and feeds off of this messiness. A multidisciplinary theoretical framework offers a suitably complex approach through which to consider the mess that emerges when patients go online to negotiate their care, as well as all the nuances and associated histories attached to those social media practices.

### Social Media Studies and Affect Theory

Following recent turns in feminist social media studies, this research approaches the habitual practices of social media using affect theory (21, 22). Affect theory provides a way of understanding how people are drawn together online, how emotions circulate through digital spaces, and what those relationships, emotions, and practices produce (23). This approach also considers the ways in which everyday habits of social media use can provide people with methods of engaging in politics or putting themselves in conversations with others that may otherwise be inaccessible to them, such as people who are not able to “take to the streets” due to debilitating endometriosis (24–27).

Affect theory also provides a tool for conceptualizing what it means to live with chronic pain, which is one of the primary symptoms of endometriosis. Neuroscientific research shows that the sensation of pain can be experienced differently depending on how it is processed or understood psychologically (28–30). The International Association for the Study of Pain defines pain as “an unpleasant sensory and emotional experience associated with, or resembling that associated with, actual or potential tissue damage” (31). The way pain comes to be felt in bodies is not just about physical damage or degeneration but is also dependent on the kinds of affects attached to that pain. This does not make the pain any less real, but only emphasizes how nuanced the experience of pain can be, particularly for people who have experienced chronic pain and are more susceptible to further pain through central sensitization syndrome (31).

Bridging the gap between the humanities and pain research, McCosker and Jackson use affect theory to explain how pain's meaning can shape how it is experienced (32, 33). Similarly, feminist scholar Ahmed uses neuroscience and affect theory to show how pain is felt and produced not only individually, but also socially (34). Although endometriosis does not always involve pain, chronic pain research is still useful for conceptualizing the way endometriosis's meaning can come to shape one's experience with it.

### Feminist Disability Studies

Endometriosis has been largely absent from feminist disability studies thus far; however, it is still an important framework for understanding the ways that endometriosis gets embodied and understood (35). Disability studies takes a social-constructionist perspective to impairment and illness, considering how such concepts such as “disability” often frame individuals as flawed in opposition to a preconceived idea of normalcy (36). Endometriosis has similarly been constructed around the idea of individual flaws, as seen through discourses of hysteria and the 20th-century idea that endometriosis was a “career woman's disease” that occurred when women delayed childbirth to have careers (10, 11, 13). Endometriosis cannot be divorced from the histories and power structures that have come to establish how it is represented today and disability studies helps frame how these external forces can contribute to how it is experienced by patients (13, 20). Disability studies also privileges the stories and lived experiences of disabled, sick, and crip people as co-creators of knowledge (37–39). A feminist disability studies framework is useful for illuminating how the knowledge and meanings that people living with endometriosis produce on social media can shift how it is understood socially and even within research.

## Methodology

### Methods

Unlike big-data analyses which use algorithms and software to collect large amounts of data such as all the posts within a certain hashtag, this study used small data practices to focus on the intentions and personal experiences of those who live with endometriosis and use social media in relation to their disease. This article follows in the practice of other feminist media scholars who use qualitative methods such as digital ethnography to consider the individuals behind the big data (40, 41). It is particularly important to the author of this study, as not only researcher but also a fellow endometriosis patient working from a disability studies approach, that the experiences of these participants are at the forefront of this research. A qualitative ethnographic approach to this topic considers not only *what* people with endometriosis post on social media but *how* and *why* they do.

### Participants and Approach

This research focuses on the platforms Facebook and Instagram as these are currently the predominant social media platforms used by those with endometriosis (16). This project was approved by the Research Ethics Board at Concordia University and all survey and interview respondents gave their consent to have their answers included in this research. The survey and interview questions as well as recruitment strategies were developed with mentorship from supervisors. To reach out to participants, a survey about endometriosis-related social media practices was shared on 5 popular private endometriosis Facebook groups with permissions from the group administrators. One-on-one interviews were then conducted with these administrators, 12 individuals who ran endometriosis-related Instagram pages, as well as those from the survey who had anomalous results, mainly two individuals whose answers were opposite to the rest of the results. Some of the interview participants were recruited using the snowball method. The interview participants ranged in age from late 20s to late 70s. Nineteen identified as women and three as non-binary or genderfluid. Eighteen of the participants lived in North America, while the others lived in Israel, South Africa, England, and Ireland. All participants were English-speaking and 7 of the 22 participants were visible minorities. All the survey participants were over 18, but no other demographic data was collected from them. All respondents identified as people living with endometriosis.

In total, the survey received 287 responses and 22 interviews were conducted. The survey took ~20 min to complete and was composed of both multiple choice and short answer questions. The semi-structured interviews were conducted over zoom using pre-structured, open-ended questions and ranged from 30 to 60 min on average with one lasting over 2 h. The 24 interview questions and survey questions were very similar, although the interviews allowed for more elaboration and included 16 additional questions about group administration and Instagram for those it pertained to. Both the interview and survey focused on asking participants about their experiences with endometriosis symptoms, diagnosis, and treatment, what the disease means to them, and why and how they use social media. The full survey and interview questions are available on the author's website for those who wish to replicate the study: https://eileenmary.net/2021/05/03/interview-and-survey-questions/.

**Figure 1:**
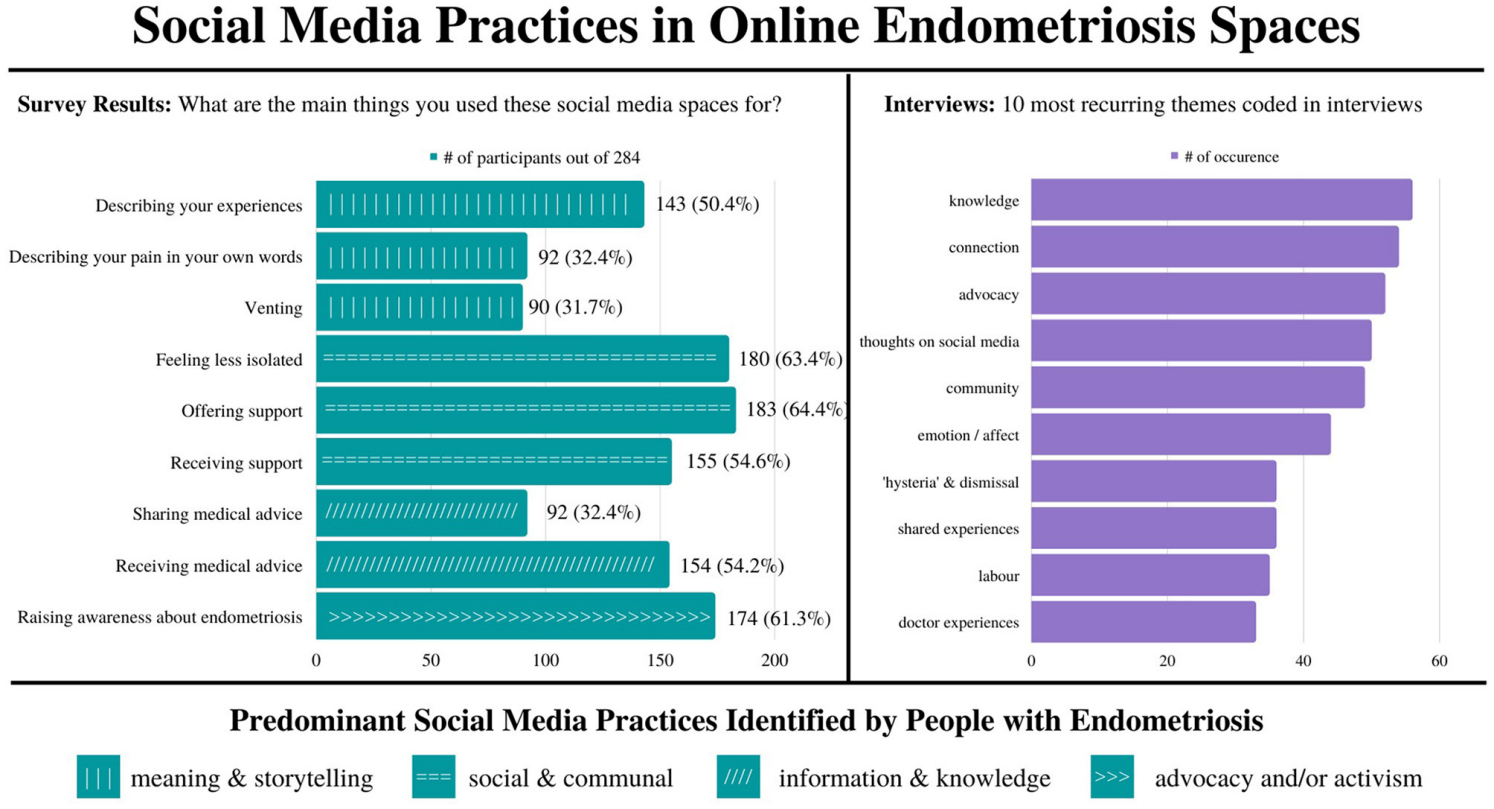
A representation of the most common social media practices in endometriosis social media spaces based on survey results and including the most recurrent themes coded in interviews.

The survey results were compiled into a spreadsheet using Google Forms where the multiple-choice results could be analyzed in graphs and the longer answer questions could be coded and analyzed thematically in Dedoose. The interviews were transcribed and put into Dedoose and then coded based on themes that emerged, as well as the predominant social media practices that were identified in the survey results (Figure 1). These themes were identified through inductive coding while reviewing the interviews based on the most prominent and recurring topics. If multiple interviewees mentioned the word community, it would be coded as “community,” but these excerpts could include differing feelings or representations of community. Multiple codes would be used per section if relevant. The total number of codes was 69, with the most discussed ones being: knowledge; connection; advocacy; reflections on social media; community, emotion, experiences and shared experiences; histories of hysteria and dismissal; labor; and doctor experiences (Figure 1).

A combination of *in vivo* coding and values coding was used (42). For the most part, participants' own words were used to determine the code names, although in the case of “histories of hysteria and dismissal,” two codes were combined into one due to crossover. “Doctor experiences” refers broadly to the stories interviewees shared about various medical experiences. Many of these codes crossed over with the social media practices identified in the survey results, such as “connection,” “knowledge,” and “advocacy.” The others, such as “labor” and “dismissal” feature in the findings and interpretations section in conversation with these broader practices.

Throughout every part of the surveys and interviews, the author was upfront about their own diagnosis with endometriosis with participants. They explained their positionality and personal investment in this research in both the calls for participation as well as before every interview. This bias was discussed upfront because, as Luka and Millette write, no research is immune from bias, whether quantitative or qualitative (40). Endometriosis research has a long history of bias toward certain voices and forms of knowledge-production, so it is particularly important to be transparent and make space for patient voices in current endometriosis research. Although limiting, this perspective was also essential to the research process, as many participants expressed feeling more comfortable sharing their answers with a researcher who understood the disease personally. The same participation might not be accessible to a researcher without endometriosis.

Due to this privileged position, measures were taken to maintain neutrality and facilitate feedback throughout the research process. Those whose survey answers differed from the majority of the responses were interviewed further to try and account for possible biased perspectives in the results, such as those who identified as less comfortable using social media. Throughout the research process, the publications born out of this research were shared with the interviewees as well as any survey respondents who provided their emails to encourage ongoing conversations and feedback. These public results have also been shared with the broader endometriosis social media network and endometriosis research networks to receive more feedback. The analysis of results was reviewed with the author's supervisor and compared to related research.

## Findings

The findings of this study have been organized based on what the survey results and interviews identified as the predominant practices people with endometriosis use on social media. These practices were then broken down into themes: meaning making and storytelling; social and communal; information and knowledge; and advocacy (Figure 1). These categories help frame what takes place in and what is produced on endometriosis social media spaces. Following the theoretical frameworks identified above, this research considers how everyday social media practices can help shape how endometriosis comes to be represented for and understood by both individuals and broader communities (43, 44). The number of survey respondents vary between results, as the participants were not required to answer every question.

### Information and Knowledge Practices

**Figure 2:**
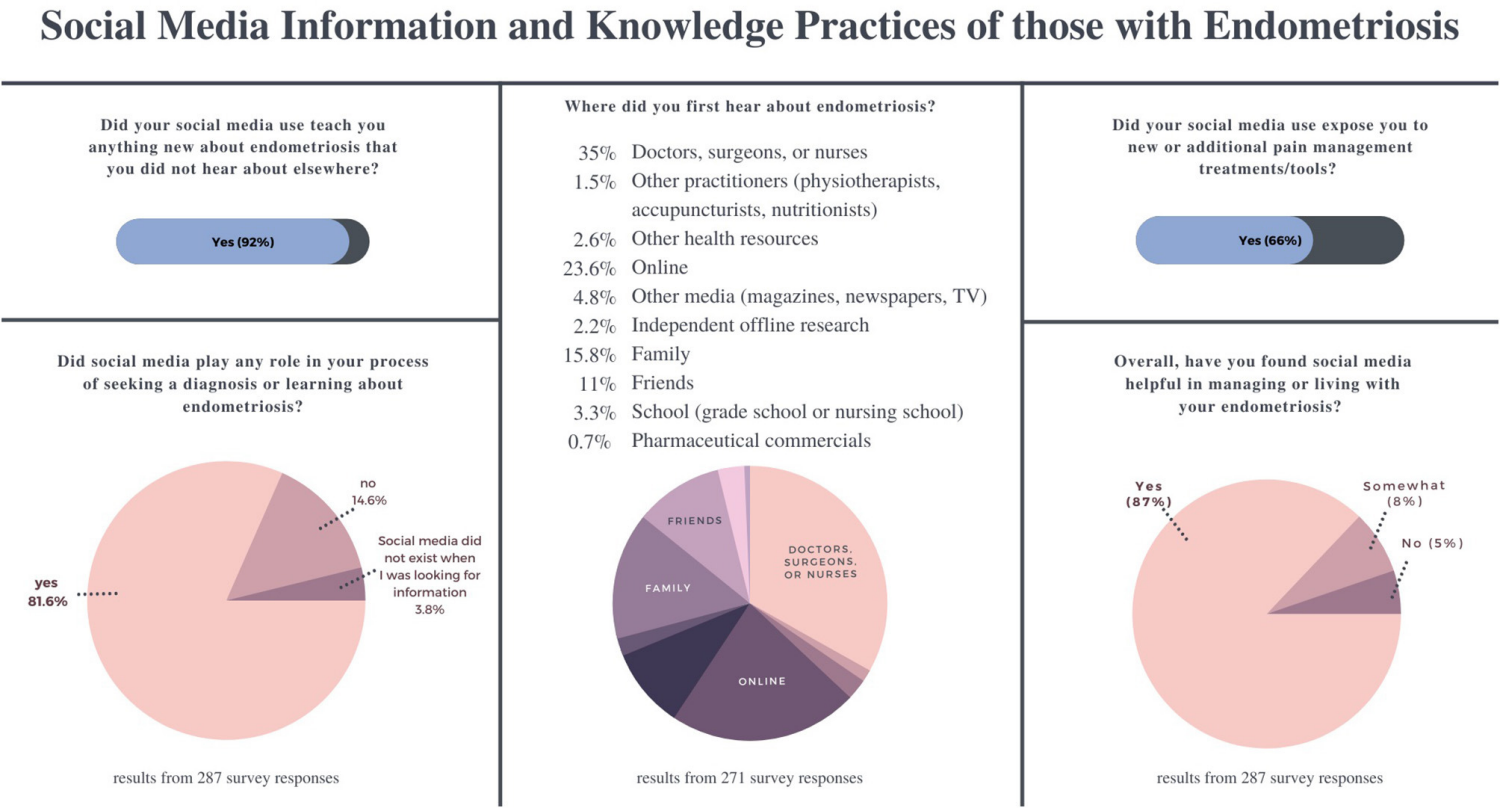
Survey results showing how people with endometriosis use social media for information and knowledge practices and how these uses have influenced their life, diagnosis, and treatment options.

When asked if social media played a role in their process of seeking a diagnosis or learning about endometriosis, 81.6% (*n* = 235) of respondents (*N* = 287) said yes, while 3.8% (*n* = 11) said that social media wasn't around when they were looking for information (Figure 2). Only 35% (*n* = 95) of respondents (*N* = 271) identified first hearing about endometriosis directly from a healthcare practitioner. 2.6% (*n* = 7) said they heard about it from other health resources, while 23.6% (*n* = 64) heard about it online from sources that may or may not have been health resources (Figure 2).

When asked if social media taught them anything new about endometriosis that they had not heard elsewhere, 92% (*n* = 264) of participants said yes. Sixty-six percentage (*n* = 189) said that social media exposed them to new or additional pain management treatments/tools and 85% said that social media had been helpful (*n* = 250) or somewhat helpful (*n* = 22) in managing or living with their endometriosis (Figure 2). This data is consistent with a number of recent studies which suggest that social media has opened up many new opportunities for information-sharing around chronic illnesses, particularly for people of marginalized genders or with stigmatized conditions (45–47). The potentials of social media for endometriosis have not been as extensively explored.

The average time between start of symptoms and diagnosis identified by my survey was 10 years—2.5 year higher than the average world estimate—suggesting that people social media use may be more common in those who have difficulty getting diagnosed. This theory is also supported by the results of the interviews, where a majority of participants identified turning to social media only after experiencing years of medical dismissal.

The benefit to Facebook and Instagram is that information is not only shared amongst many people, but that individuals can also save time by learning from one another's experiences. One interview participant described how this saved her time in her own treatment process.

I wouldn't know [that pregnancy didn't treat endometriosis] if it wasn't for all these different Facebook groups, Instagram profiles […] I think I had to see other people in different parts of that journey and what they were going through to really help myself make those decisions. […] I skipped through a lot of that initial hair pulling frustration because I had people pointing me in the right direction.

Another participant described how she has seen social media empower people with endometriosis to ask for more from their practitioners:

Women now have gone from maybe trusting their doctors implicitly to starting to question them and with the use of social media in particular […] they are now able to go in and see themselves as equal to their medical team as well. They're able to negotiate a wee bit more and certainly in Ireland they're asking for referrals outside of the country because we don't have a lot of doctors who are able to help.

Survey respondents and interviewees both talked not only about their experiences being dismissed for their symptoms, but also for using social media for information. As one interviewee said:

I did my own research and I know doctors hate when patients consult 'Dr. Google' but I wasn't getting any help anywhere but so I was just doing my best to put pieces together on my own.

The survey respondents and interviewees held differing opinions on which sources were the most reliable and, as one interviewee said: “what one person considers misinformation, another person considers accurate.” Despite the misinformation and complexity of information-seeking on social media, the survey and interview results still showed that, overall, it was useful for many of the respondents.

### Social and Communal Practices

**Figure 3:**
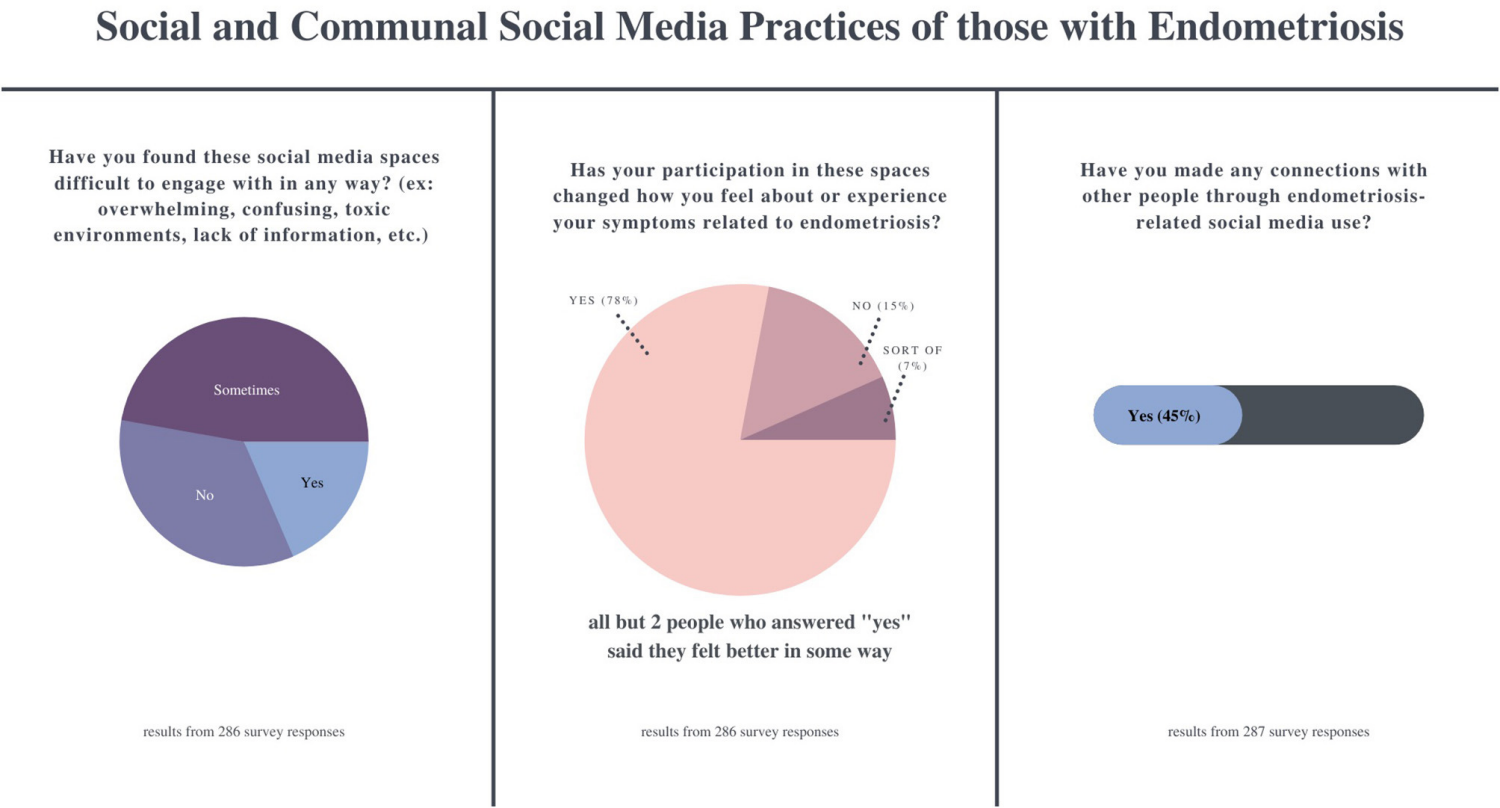
Survey results illustrating the social and communal practices of people with endometriosis on social media.

Both the survey respondents and interviewees commonly expressed that online support groups and Instagram spaces made them “feel less alone.” Seventy-eight percentage (*n* = 223) of participants (*N* = 286) said that participating in endometriosis social media spaces changed how they felt about or experienced their symptoms, with an additional 7% (*n* = 20) saying it “sort of” did (Figure 3). Forty-five percentage (*n* = 129) of participants (*N* = 287) said they had made connections with others through their endometriosis social media use. Those who did described feelings of community, connectedness, and friendship based on shared experiences:

There's a, I would say unspoken but it's also spoken, bond that I think just happens. It happens with anyone with shared experience that involves being not only in so much pain, but dismissed, marginalized in our own ways. If you tell me you have endo, I know what that means and I don't care who you are, I'm here, what do you need?

I feel like there is a really strong community, not here in Israel specifically, but around the world […] because everyone experiences basically the same thing. At different levels, but people know what you mean.

I've made some really good friends now in the last 6 months and there are people I check in with more online as opposed to real life because they get it and you can kind of message people and go “I've had a really bad day” and you don't have to explain why […] which is really lovely and I think that has really helped my mental health too, just knowing that if I need to talk to someone or if I need to vent about something generally people [will reach out].

I have found support simply through the realization that there are many people who experience these symptoms than I thought, and that there is a valid explanation for the pain. It has been very validating to hear other people's experiences, particularly when it comes to being dismissed by medical professionals when seeking treatment.

When you tell someone you have endometriosis and they tell you back, it's like we're already friends.

Many survey respondents specified that they only use social media for information, not connections. Others explained that they felt some connection with others but had not made any friendships or more substantial relationships. In the interviews, participants often used the words “community,” “tribe,” “sisterhood,” or “brotherhood” to describe the experience of participating in online endometriosis spaces, despite also describing how the spaces can sometimes be unpleasant or unwelcoming, particularly for those from marginalized backgrounds such as visible minorities or those who do not identify as cisgender women (those assigned female as birth who live as women).

The survey and interview respondents were also transparent about the fact that online endometriosis spaces can be difficult to inhabit sometimes. When asked if they found endometriosis social media spaces to be difficult to engage with in any way (ex: overwhelming, confusing, toxic environments, lack of information, etc.) 47.2% (*n* = 135) survey participants said sometimes, while 18.5% (*n* = 53) said yes and 34.3% (*n* = 98) said no (Figure 3). The interviews highlighted these challenges:

I think this is my first time saying it out loud, but […] when I mentioned seeing sick people and all the suffering […] I started to wonder if I was sick enough to be a part of this group. I do have a lot of good days; I do have days where my body is kind to me and I have peace on that day. […] So I kind of have to learn to be kind not myself and say, “it's okay that you are okay.” You don't have to be sick or unwell or in pain all the time to relate to other people in this space.

I don't know, part of me doesn't want to be part of [many of the groups] because I don't see those spaces as safe places for non-binary or trans people and I think the communities need to be more actively focused on changing the language. And I don't think that's just the fault of cis-gendered women who have the disease, I think it's also the fault of the medical community in framing endo as a woman's disease.

I've seen queer people post in those spaces about what needs to change to make those spaces safe for them and a lot of especially white, cisgender women being like “well, get out of this space then.” Like really toxic, gross stuff.

I didn't find anyone that was my color that was talking about it. And you know, within our community, we don't really discuss the health issues that we deal with […] I really wanted to find […] a community for other people that are African American just to see if we could compare notes.

Because people have to self-advocate with endo so much, most people do a lot of research on it. [But] people are also constantly learning about endo for the first time […] Because of this, I think there's often a clash in social media spaces because it seems that the well-researched folks get very annoyed when people who are just beginning their journey ask questions.

The perspectives of the respondents were often conflicting. For example, while one interview respondents said that there needs to be room for hard feelings in endometriosis social media spaces, another said that the emotional intensity of the spaces caused them to feel worse about their own condition:

I feel like there needs to be a space for people to share the hard things because this is a hard thing we're going through. And there are no easy answers and feeling hopeless or frustrated or upset is like the natural human response to being in a situation that's awful.

It came to a point that I needed to just put those pages on mute so that I could go to the page when I was feeling like I needed to find some information or just completely unfollow it because it wasn't good for my own mental space to just keep seeing how hard it is for people to live with this thing. So I had to kind of just separate myself from social media sometimes because it did become overwhelming and toxic.

Although some of the interview and survey participants found the social and communal aspects of social media to be very important and/or beneficial, this was not the case for all participants. While some found the social and communal aspects of endometriosis social media space difficult or overwhelming to engage in, others were just more interested in focusing on information and knowledge-sharing.

### Meaning-Making, Storytelling, and Advocacy Practices

The other common practices on endometriosis social media spaces are meaning-making, storytelling, and advocacy practices. Beyond the value of information-sharing, education, and social supports, social media also offers a place where patients can make their endometriosis visible, describe it in their own terms, and even contribute to new cultural understandings of the disease (48). 50.4% (*n* = 143) of survey respondents (*N* = 284) described using social media to “describe their experiences,” with 32.4% (*n* = 92) saying they used it to “describe their pain in their own words” and 31.7% (*n* = 90) using it for “venting.” 61.3% (*n* = 174) said they use social media to raise awareness about endometriosis (Figure 1).

The interviewees described the different ways that storytelling, meaning-making, and representation on social media was useful to them:

I'm a writer so being able to write things out [on social media] is very helpful and I also have memory problems sometimes so being able to go back and reread things and say I was in the worst pain fog when this conversation happened, like what were those tips and what was being shared and having a kind of record for that.

If I was having a bad day, I could say to my boyfriend, just read my [Instagram] post from today because that's how I'm feeling and he would go, oh that makes sense. But now […] I have so many people going “oh my god, it's so nice to know that I'm not alone in this” and “it's funny and you shouldn't be laughing but it's nice to laugh at it.

” So that account really started as just a diary for me, just to therapeutically be able to get it out and it wasn't even for connection at that point, it was just to get it off my chest, like this is what I'm dealing with, this is what my day-to-day is like. And then once I had surgery and I had more to share about my updates I had a lot of people asking questions and then it turned into more of an education resource.

The day that I came out with my diagnosis I posted a picture, I'm sure you've seen it 100 times yourself with the surgery scars and the plasters over it. I posted a picture of that and explaining my story. Prior to my surgery I had posted about the fact that I was going into surgery to have a cyst removed and I thought I should tell you all because nobody speaks about this stuff so I'm telling you about it. […] I can't count the number of times people have said to me: “I don't feel alone anymore. Thank you for sharing your story.” And you know that's a virtual connection. That's someone would have made through something I would have spoken about.

One of the interviewees also described how online patient representations can also be useful for doctors, as well as for patients by capturing the qualitative aspects of pain which is the clinical pain scale does not account for. For example, in the interview she describes the experience of diaphragmatic endometriosis:

Someone says ‘I feel like I have an elephant sitting on my chest and I’m trying to blow up a flat balloon.’ Maybe you don't know what it's like to have an elephant sit on your chest, but you know what it's like to blow up a flat balloon and how hard it is.

Some of the interview respondents also spoke about how social media inspired them to engage in advocacy and awareness raising:

I'm a late comer to the endo community and jumping into the advocacy (it's not that I didn't have the condition, it's that we weren't really aware of that being something to be looked at) and I think part of that makes me mad because there are others like me, in my age group, that have gone decades that have struggled with the disease, really traumatic diseases, people that are isolated incidences, and it was always under the radar and I look at these people now and some of them, they had their whole life ahead of them and it just changed all that.

And I mean when I did start speaking about [endometriosis online] I had no idea what to expect because I had never been an advocate for anything in my life before this time. So I didn't know what could have been coming but I think I got the best response because so many people could relate to me.

At the beginning [when] I was starting [my Instagram] it was honestly it was mainly just for me. I mean I wanted to help other women and other people honestly but I was also like, I had no idea what's going on so I'm just going to start talking loudly and then hopefully someone will hear me and then I'll get help. I mean the reason I do it now, which I say over and over, is just so no woman thinks that they're alone in their pain. That's basically just the bottom line of why I do it now. Because I felt so alone and I felt no one knew what my pain was, no one understood my pain and I was, I want to be that person for someone else that does understand.

The way that participants understood and described their endometriosis both online and in the surveys and interviews was multiplicitous. In both the survey and interviews, participants were asked the open-ended question, “What does ‘endometriosis’ mean to you? Has this changed over time?” Some answered with clinical descriptions, some explained their symptoms, others were more metaphorical. While some answers focused on debilitating symptoms, others spoke of hope and growth. For some, endometriosis took on a kind of personality, described as “sadistic” or “controlling.” For others, it was not only a disease, but a way of understanding the world, as shown by the answer “[endometriosis] has taught me access to care is unequal.” One participant wrote, “[i]t's something that's forever a part of my life… but won't define it.” Another said “it means losing out on my ‘timeline’ for my own life.” The most persistent things that people talked about were pain, their lives, and the time endometriosis takes away from them. The most common words and themes that appeared in the answer to this question were symptoms (“fatigue,” “pain,” “cramps,” “heavy [bleeding],” “nausea”); temporality (“time,” “years,” “always”); actors (“doctors,” “medical,”); challenges (“struggle,” “trying,” “work”; “without,” “help”); body parts (“organs,” “endometrial”). In order to fully try and grasp the complexity of what endometriosis can mean to patients, all of these different aspects (individual, physical, structural, systemic) need to be considered. These nuance answers reflect the complex representations that are shared in endometriosis social media spaces every single day.

## Discussion

It is clear from looking at the varied social media practices of people with endometriosis that the illness cannot be easily summarized as just one thing. This complexity and nuance are part of what makes endometriosis social media spaces so significant. There are many different players at work on social media, and many differing viewpoints and experiences. Currently, social media offers a subjective, qualitative, and complex view of endometriosis that is rarely being represented clinically or even within research. Simply through posting and creating on social media, people with endometriosis are influencing how the illness gets talked about and challenging the simplistic ways that it has been addressed thus far with their experiences, connections, and information-sharing. In this section, the above findings will be explored by again breaking them down by practices:

### Information and Knowledge Practices

The survey and interview results reveal a predominant communication and information gap between doctors and patients in the case of endometriosis. This trust gap has been something that has continually been acknowledged in research around stigmatized and gendered illnesses (49, 50). The kinds of information and knowledge produced in endometriosis social media spaces is not simple or straightforward and, as other scholars have explored, social media is rife with misinformation (16, 19).

Instead of becoming a further barrier to accessing care, with the right approach and support from practitioners, information-seeking and knowledge-sharing on social media could help improve time to diagnosis. A recent systematic review shows that seeking health information online can actually improve the patient-practitioner relationship when both parties talk about it openly and without judgment (51). These survey and interview results also suggest that if patients can access information from medical professionals sooner, they may not turn to social media for information as extensively.

The information-seeking practices of people with endometriosis on social media also show that patients value more than just scientific or medical knowledge. Shared experiences also play an important role and, although experiential knowledge tends to be considered less rigorous than biomedical knowledge, the survey results and interviews reveal that it is very valuable to many of those who live with endometriosis. In fact, by sharing their experiences online, people with endometriosis are helping create new forms of knowledge that others can draw on to understand their own experiences.

### Social and Communal Practices

Research has shown that online support groups can be helpful for improving the overall wellbeing and quality of life for people with chronic illnesses through reducing isolation, improving feelings of support and community, and increasing patients' confidence interacting with their healthcare professionals (45, 52–54). However, online support groups and illness spaces can increase the risks of conflict, developing maladaptive coping strategies, or experiencing emotional overwhelm (17, 45, 52). These conflicting results were also reflected in the survey results and interviews of this study, where respondents expressed benefits but also challenges in connecting with others online.

Existing endometriosis research tends to be more accepting of using social media for support and community than information-seeking, however studies do not always acknowledge the complexities of these spaces (6, 16, 17, 55). The survey and interview responses show how conflicts that emerge in these online spaces often parallel the conflicts that exist in the societal and biomedical construction of endometriosis, such as longstanding prejudices, stigmas, and medical debates. The emotional toll of endometriosis, which is not always addressed clinically, also takes up space in these online communities and can drive the kinds of conversations these spaces produce. As feminist affect scholar Sara Ahmed writes, “emotions in their very intensity involve miscommunication, such that even when we have the same feeling, we don't necessarily have the same relationship to the feeling” (34). The contradiction of endometriosis spaces is that they both thrive off of shared experiences and shared feelings, but that these experiences inevitably contain differences, nuances, and conflicts.

Although, as the previous section showed, it is important not to dismiss the social media practices of people with endometriosis, it is also important that clinicians and researchers understand the full extent of these groups before referring their clients to them for emotional support. The social and communal aspects of online social media spaces reflect and exacerbate the conflicts and inequalities that already exist in endometriosis care. While social media can be beneficial, and while patients will continue to use these platforms if they don't have access to better alternatives, the complexities of these spaces should not be overlooked.

### Meaning-Making, Storytelling, and Advocacy Practices

There has been extensive research and writing across disciplines on the challenges of communicating pain and illness, whether between a patient and practitioner or more broadly (56–59). Chronic pain and illnesses involve biological, psychological, cultural, and social factors that can make it difficult to assess quantitatively (60, 61). Despite the need for an interdisciplinary approach to pain, throughout Western medicine it is still common for many practitioners to use the “pain scale” in clinical practice, where patients are asked to label their pain on a scale quantitatively from 1 to 10. This tool has been critiqued for its subjectivity and, although other alternatives exist, they are not as commonly practiced (62, 63). Despite the prevalence of chronic pain and illnesses, people living with them are likely to experience disbelief and invalidation from their practitioners, which can cause isolation, depression, and emotional distress (64, 65).

Although endometriosis does not always involve chronic pain, it is one of the most common symptoms [experienced by at least 92% (*n* = 261) of my survey respondents], and research about pain communication provides a useful way for understanding how endometriosis gets represented, mediated, and recreated online. Elaine Scarry's formative work, *The Body in Pain*, is particularly useful for framing how pain's seeming unrepresentability can “unmake” an individual subject's world, while also “making” new worlds both despite and because of this unrepresentability. Pain's lack of referential content, its unfathomability, can destroy language, but this objectlessness also “gives rise to imagining” and new forms of meaning-making (57). For many endometriosis patients, the pain can be so severe that it renders a person unable to speak or move.

In contrast, the representation of endometriosis on social media is almost incessantly focused on *making that pain visible*. This visualization practice is sometimes done through language in Facebook posts or Instagram infographics, where pain is put into words and metaphors. It is also often represented visually online: sometimes through drawings; painting physical wounds on the outside of the body; or photography of a vulnerable moment, such as a person recovering from surgery or kneeled over in pain. The externally invisible, but full body, nature of the disease has also been represented through Instagram campaigns such as #IAmExtraNotRare or #ThisIsEndometriosis, where participants are asked to visually represent endometriosis on their body with graphics or by writing on their skin.

Social media also allows those who do not explore representational practices themselves to benefit through seeing other people's posts and potentially recognizing themselves in those shared experiences. Patient narratives, descriptors, or metaphors can also be useful for showing how people with endometriosis experience, feel, and understand their pain. For example, in a study by Stella Bullo and Jasmine Heath Hearn, 21 women described their endometriosis with metaphors that represented it as an external agent controlling their experiences, showing that they generally felt a helplessness and lack of control surrounding their disease (66). While patient representations are never perfect examples of endometriosis, the intense, affective, or fragmented metaphors that people use to describe their endometriosis can often reveal the emotional and physical toll of the disease better than the description itself. As Dolmage writes about disability storytelling and rhetoric, imperfect narratives like these have value—“meaning actually springs forth from gaps and flaws and mistakes” (67). The value of online endometriosis representations is not just about what is created, but about what the practice of representation itself reveals.

In this way, patients who post on social media create their own research networks of endometriosis outside of academic or medical institutions. But these networks can be complicated and unreliable. These storytelling practices have also been picked up by endometriosis organizations, pharmaceutical companies, and practitioners, who sometimes share patient narratives to add an impact to their own posts or sell products. As one interviewee describes, sharing personal stories gets her the “greatest engagement” on Instagram. While some may be sharing their stories for their own benefit, others may be using it as an advertising tactic. The online network of endometriosis stories is unregulated and the intentionality behind posts can be difficult to untangle for people just scrolling by.

The varied and sometimes contradictory meanings of endometriosis that survey and interview respondents shared speak louder when put in conversation with one another. The *social* nature of social media allows for some of endometriosis's complexities to be more thoroughly explored. Scholar Anthony McCosker draws together the social sciences and neuroscience to argue that pain cannot be defined by only one body. As he writes,

The affective force of pain is located not simply within the perceiving subject, nor the object that ‘initiates’ sense perception, nor in the impulse striking out between cells in afferent synaptic chains coursing through the body, nor at the synaptic interference or within the nerve cells themselves at the site of a wound […] Rather the affective force of pain lies in the complex interchange of any and all of these elements, along with others, through which bodies act upon one another and in relation to one another within an encounter (32).

Although pain and illness are always embodied individually, they are also structured through relations and cannot be divorced from the power structures that come to situate their meanings, feelings, and affective dimensions. Endometriosis comes to be felt through complex relations within ourselves, but it is even further constructed through social relations, the support of others, our experiences in medical institutions, our conceptions of ability and disability, our understandings of pain, our desire for productivity, and our media practices. Endometriosis, in all its relationalities, cannot be measured simply by numbers or even symptoms and to do so would be to do it a great disservice. Social media offers a glimpse of what a more complex representation of endometriosis might look at, but the people living with this disease deserve safer, more regulated spaces to explore this self-expression.

### Implications

Endometriosis social media practices create an important, but messy, archive of the disease. This archive is built collectively through the narratives of many different people with the disease, practitioners, pharmaceutical companies, businesses, and more. The complexities of these crossovers only enhance the political density of these spaces and the potential resistance people with endometriosis engage in by participating. In a world where neither online or offline space are safe from healthcare inequalities, many with endometriosis are using social media to try and create an alternative.

As Gonzalez-Polledo writes of chronic pain communication on Tumblr, “in social media pain is reframed as a political issue as it is transformed from an individual, potentially disabling event that has the capacity to put life on hold to an inherently social, actionable, collective, *issue*” (59). Those living with endometriosis who engage collectively in these spaces “resist epistemic injustice and create inhabitable pain worlds” (59). By paying attention to the social media practices of those with endometriosis, we get not only “pain worlds,” but *endometriosis* worlds, where the present and future of the disease can be reimagined. While this online engagement, and these endometriosis worlds, may not always change the dominant narrative, if we consider the long history of endometriosis—including the continued dismissal and mistreatment of patients and the influence of sexism, racism, and classism—even the sheer volume of patient voices on Facebook and Instagram is already an significant change. People with endometriosis have a place to connect, discuss, and learn from one another in ways that have never before been possible and this historical relevancy should not be underestimated.

It is important that practitioners and researchers going forward understand the amount of labor that people with endometriosis who use social media must conduct in order to access information, care, and support online, as well as raise awareness about the disease. Social media helps reveal the gaps in care for endometriosis patients and, although this article shows that it can be beneficial to some patients, it not a long-term solution. As Dusenbery writes in her book *Doing Harm*, “[w]hile some patients may *want* to be partners in their medical care, and the Internet has certainly made it easier for some patients to educate themselves, not all women have the vast resources require to become ‘empowered patients.’ And doing so should not be mandatory” (49). Understanding the realities that face so many people with endometriosis and why they might turn to social media can help practitioners and researchers have more productive conversations about patient social media use and patient-centered care.

### Limitations and Recommendations for Future Research

The survey and interview participants for this research were recruited through social media so, although participants had varying degrees of usage, this study does not account for people with endometriosis who do not use social media at all. For a fuller picture of the role social media plays in the broader population of endometriosis patients, future research should recruit through other sources, such as patient groups and clinics. This study also focuses predominantly on English-speaking social media spaces with most participants coming from North America. Future research is needed to address this gap.

A broader method of analysis such as data-scraping could better inform on the wider range of post styles that appear in endometriosis spaces. This article is limited to self-reporting from survey respondents and interviewees who have endometriosis and therefore does not consider the many other people involved in online endometriosis spaces, such as clinicians, businesses, and caregivers. Due to the complexity and time-consuming nature of ethnographic research, this study was only able to cover a limited number of participants and the snowball method of recruitment for interviews could have resulted in more homogenous responses. Future researchers might consider using other qualitative methods such as focus groups to hear from a wider range of people. In particular, it would be beneficial to conduct focus groups with endometriosis patients so that they could workshop the interview questions and research methods before the research period using their lived experience. Future endometriosis research should do more to collaborate with patients as co-researchers/co-creators and, although this study aimed to emphasize patient voices, it would have benefited from having more patient collaborators contributing to and shaping the research process itself.

Ideally, future researchers in this area should also establish a larger and more interdisciplinary research group so that more data can be collected, and the results can be more thoroughly and complexly analyzed. A larger research team would not only help mitigate bias but could also be useful for addressing the accessibility needs that come up when researchers are themselves patients, as is the author of this article.

## Conclusion

This research highlights the importance of not oversimplifying the experience of living with endometriosis. By looking at the social media practices of people with endometriosis, the disparities in care, histories of medical neglect, and complexities of life with chronic pain and illness are revealed. Significant archives of experience and patient-produced knowledge are available on social media, and these should not be dismissed or ignored. Although social media use comes with complexities and challenges, fully understanding why patients go online and what they use these spaces for can help practitioners and researchers better understand what people with endometriosis need from their care, such as validation and support, reductions to diagnosis, better access to accurate information, emotional support, and clearer representations of endometriosis in medicine and popular culture. This article is a first step toward validating and analyzing the social media practices of people with endometriosis so that future researchers and clinicians can use these findings toward shaping patient-centered futures.

## Data Availability Statement

The raw data supporting the conclusions of this article will be made available by the authors, without undue reservation.

## Ethics Statement

The studies involving human participants were reviewed and approved by Concordia University Human Research Ethics Committee. The patients/participants provided their written informed consent to participate in this study.

## Author Contributions

The author confirms being the sole contributor of this work and has approved it for publication.

## Funding

The author's research was funded *via* the Joseph-Armand Bombardier Canada Graduate Scholarship (CGS) Doctoral Social Science and Humanities Research Council of Canada (SSHRC) Grant.

## Conflict of Interest

The author declares that the research was conducted in the absence of any commercial or financial relationships that could be construed as a potential conflict of interest.

## Publisher's Note

All claims expressed in this article are solely those of the authors and do not necessarily represent those of their affiliated organizations, or those of the publisher, the editors and the reviewers. Any product that may be evaluated in this article, or claim that may be made by its manufacturer, is not guaranteed or endorsed by the publisher.
